# True and false positive rates for different criteria of evaluating statistical evidence from clinical trials

**DOI:** 10.1186/s12874-019-0865-y

**Published:** 2019-11-27

**Authors:** Don van Ravenzwaaij, John P. A. Ioannidis

**Affiliations:** 1 Department of Psychology, University of Groningen, Grote Kruisstraat 2/1, Heymans Building, room 169, 9712 TS Groningen, The Netherlands; 2 Departments of Medicine, of Health Research and Policy, and of Statistics and Meta-Research Innovation Center at Stanford (METRICS), Stanford University, Stanford, USA

**Keywords:** US Food and Drug Administration, *p*-values, Strength of Evidence, Bayes Factors

## Abstract

**Background:**

Until recently a typical rule that has often been used for the endorsement of new medications by the Food and Drug Administration has been the existence of at least two statistically significant clinical trials favoring the new medication. This rule has consequences for the true positive (endorsement of an effective treatment) and false positive rates (endorsement of an ineffective treatment).

**Methods:**

In this paper, we compare true positive and false positive rates for different evaluation criteria through simulations that rely on (1) conventional *p*-values; (2) confidence intervals based on meta-analyses assuming fixed or random effects; and (3) Bayes factors. We varied threshold levels for statistical evidence, thresholds for what constitutes a clinically meaningful treatment effect, and number of trials conducted.

**Results:**

Our results show that Bayes factors, meta-analytic confidence intervals, and *p*-values often have similar performance. Bayes factors may perform better when the number of trials conducted is high and when trials have small sample sizes and clinically meaningful effects are not small, particularly in fields where the number of non-zero effects is relatively large.

**Conclusions:**

Thinking about realistic effect sizes in conjunction with desirable levels of statistical evidence, as well as quantifying statistical evidence with Bayes factors may help improve decision-making in some circumstances.

## Introduction

For over half a century, the US Food and Drug Administration (or FDA) has been one of the primary regulatory agencies worldwide when it comes to providing quality control of new drugs and biologics, or medications, for clinical use [1]. Included in the many responsibilities of the FDA is the endorsement of new medications. Endorsement of new medications is a rigorous process that follows many stages of multiple clinical trials. Data in each of these trials is evaluated, typically independently, by conducting some form of statistical inference. Typically, some null hypothesis is postulated, stating that the new medication is ineffective (more precisely, works similarly to a placebo medicine without any active component). This hypothesis is then evaluated using a Null Hypothesis Significance Test, which quantifies the probability of obtaining data with an effect at least as strong as that in the data at hand using a *p*-value*.* The effect is typically considered statistically significant when a two-sided test yields a *p*-value lower than 0.05 and the effect is in the expected direction [2, 3]. In order to mitigate the risk of incorrectly concluding effectiveness, the FDA typically requires “… at least two adequate and well-controlled studies, each convincing on its own, to establish effectiveness.” (p. 3) [4].

In previous work [5], we showed with simulations that using a criterion of at least two statistically significant trials, quantified by *p*-values lower than 0.05, leads to inconsistent strength of evidence in different circumstances. In particular, we demonstrated that for cases when many clinical trials were conducted out of which exactly two were statistically significant (say, 2 out of 5), the decision to endorse can be much improved by quantifying the evidence of all trials (not just the statistically significant ones) and a natural, convenient way to do this would be by using Bayes factors [6]. In scenarios where blind application of a criterion of two significant trials would always lead to endorsement, Bayes factors would lead to different inferences depending on the presumed underlying population effect.

The scenario of endorsement of medication following a number of registered clinical trials (RCTs) of which only a minority has been statistically significant at a *p*-value threshold of < 0.05 is not uncommon for certain groups of medication. For instance, in the field of antidepressants, citalopram was endorsed following 5 RCTs of which only 2 were statistically significant (the non-significant trials had *p*-values ranging from 0.224 to 0.964). Sertraline similarly was endorsed following 5 RCTs of which only 1 (or 2, if one counts a trial with three ‘sub-trials’ of which only one was statistically significant) was statistically significant (the non-significant trials had *p*-values ranging from 0.21 to 0.87). The antidepressant mirtazapine was endorsed following 10 RCTs of which 5 were statistically significant (the non-significant trials had *p*-values ranging from 0.19 to 0.49). Finally, bupropion was endorsed after 3 RCTs, only one of which was statistically significant (the non-significant trials had *p*-values ranging from 0.16 to 0.53). More details of the FDA registered data of these clinical trials for these medications can be found in [7] (see also [8] for a Bayesian meta-analysis).

The endorsement of new medications is certainly not a purely statistical process alone and it is not using simply an automated count of statistically significant results. Aside from numerically evaluating the efficacy of the main outcome, usually careful thought is given to potential side effects, the urgency of the availability of a new treatment, and a qualitative evaluation of the design, conduct and findings in each of these RCTs. A nuanced assessment of the global evidence takes place before reaching licensing decisions. Some guidance exists in this regard, but details may be applied differently in each case and translating results from clinical trials into a licensing decision can be convoluted. Nevertheless, the statistical evaluation is an important centerpiece of the endorsement process, so care should be taken for this component to evaluate the available evidence as optimally as possible, before other considerations are superimposed.

There are many ways one may define optimal evaluation of the available evidence. One could seek to minimize the endorsement of ineffective medications, or *false positives* (the equivalent of maximizing *true negatives*: not endorsing ineffective medications, see e.g., [9]). An advantage of such a criterion is that most of the medications that become available to the market will work. However, setting too stringent a criterion of this type might lead to many effective medications not passing the bar for endorsement, leading to patients being deprived of potentially effective treatment. A different criterion would be to maximize *true positives* (the equivalent of minimizing *false negatives*). Such a policy would prioritize making all working medications available in the market, at the cost of having some ineffective medications pass through as well. Within any kind of statistical evaluation tool, these two criteria typically trade off against one another, but across different statistical inferential methods, dominant approaches are theoretically possible, where one method offers higher true positive rates without necessarily offering a higher false positive rate.

In this paper, we examine the consequences of using a statistical evaluation criterion of two significant trials for the true and false positive rates in the specific case where exactly five trials were conducted (e.g., a situation seen in the citalopram scenario). We compare these to two alternatives: quantifying all available evidence with confidence intervals based on a summary effect size measure obtained from meta-analysis, and with Bayes factors. For both the null hypothesis significance test and the Bayesian test, choices need to be made for the threshold of evidence that would trigger endorsement. The null hypothesis test traditionally uses an *α* of 0.05, but recently a lower *α* of 0.005 was proposed [10]. No consensus exists for the Bayesian threshold of endorsement, although criteria of Bayes factors of 20 [11] and 10 [12] have been proposed as a threshold for strong evidence. We conducted simulations, varying the number of clinical trials that were conducted, the distribution of true effect sizes in the population, the number of participants per randomized control, the statistical evaluation method, the threshold for quantifying statistical evidence, and the threshold for an effect being considered *clinically meaningful*. We evaluated the outcomes by looking at the true positive and false positive rates simultaneously.

## Method

We conducted simulations for the scenarios of 2 clinical trials, 3 clinical trials, and 5 clinical trials. For each of these scenarios, we conducted four sets of simulations. For every set, we generated 8000 data sets of 2, 3, or 5 clinical trials each (a total of 16,000, 24,000, or 40,000 trials, depending on the scenario). All of the data sets were intended to mimic two–condition between–subjects experiments with an experimental group and a control (e.g., placebo) group. The four sets of simulations differed in the distribution of true population effect sizes between the two groups. For all sets of simulations, there was a distribution of non-zero effect sizes, normally distributed with a mean of 0.4 (a small to moderate effect) and a standard deviation of 0.13. For the first three sets of simulations, there additionally was a number of zero-effects. The sets differed in the relative frequency of non-zero and zero effects: the first set of simulations had a 25% occurrence rate of null effects, the second set of simulations had a 50% occurrence rate of null effects, the third set of simulations had a 75% occurrence rate of null effects, and the fourth set of simulations did not include any null effects. These different numbers reflect different rates of ‘a-priori optimism’ of the occurrence of true effects (25, 50, 75, and 0%) among medications subjected to stage III trials that try to secure licensing. Throughout the paper, we work with effect sizes in standardized form to facilitate computations and allow for comparison across results. For specific cases, effect sizes can be re-expressed in absolute form by multiplying by the standard deviation. Of note, standardized effects of the same magnitude may be clinically actionable in some cases, but not in others, depending on what the standard deviation is, what outcome they pertain to, and what the risk-benefit involved is.

Therefore, our simulations are:

*p*_*123*_ ~ *N* (0, 1)

*Set 1: 25%* of *e*_*1*_ ~ *N* (0, 1); 75% of *e*_*1*_ ~ *N*(*δ*, 1)

*Set 2: 50%* of *e*_*2*_ ~ *N* (0, 1); 50% of *e*_*2*_ ~ *N*(*δ*, 1)

*Set 3: 75%* of *e*_*3*_ ~ *N* (0, 1); 25% of *e*_*3*_ ~ *N*(*δ*, 1)

*Set 4: e*_*4*_ ~ *N*(*δ*, 1)

*δ* ~ *N* (0.4, 0.13)

where *p*_*123*_ indicates simulated data for the placebo groups in all sets of simulations; *e*_*1*_, *e*_*2*_, *e*_*3*_, and *e*_*4*_ indicate simulated data for the experimental groups in the first, second, third, and fourth set of simulations respectively; and where *δ* indicates the population effect size for a given iteration. The notation ~*N*(*μ*,*σ*) indicates that values were drawn from a normal distribution with mean and standard deviation parameters given by *μ* and *σ*, respectively.

The choice of parameters is consistent with empirical data. For example, one empirical assessment of 743 randomized trials on cancer, neurological and other diseases estimated an average treatment effect of the new treatment versus the old or placebo corresponding to a hazard ratio of 0.9 (i.e., a small effect) [13] and it is assumed that about half of the treatments that reach that stage of testing for these diseases may have some efficacy [14]. Among the 18 oncology drugs licensed by FDA between 2000 and 2011, the median hazard ratio was about 0.7, i.e. a moderate effect [15]. However, early trials may have inflated effect sizes. Large-scale evaluation of about a quarter of a million trials suggests that large effects may be seen in some small trials, but typically do not hold up upon further evaluation; thus most treatment effects of effective interventions are likely to be small or modest [16]. In the case of antidepressants like citalopram, the estimated standardized treatment effect in large meta-analysis is about 0.3 [17].

For each distribution of effect size simulation set, we ran four different kinds of number of participants per group in each trial: *n* = 20, *n* = 50, *n* = 100, and *n* = 400. These numbers are typical of what is seen in much of the biomedical literature. For example, the mean sample size (both arms combined) of the 743 randomized trials discussed above was 400 participants (200 per arm) [13] with substantial variability across different interventions and the average randomized trial published in 2000–2006 had a median sample size of 80 (both arms combined) [18]. For anti-depressants, randomized trials with between 20 and 50 participants per arm are not uncommon, and in some cases arms with fewer than 20 participants are analyzed by FDA [7].

Thus, to sum up, our simulations varied along the following dimensions:

1. Prevalence of true null effect size: 25, 50, 75, and 0%
2. Number of participants per group: 20, 50, 100, and 400

This resulted in a total of 16 types of simulations. We replicated each simulation type 2000 times. Every individual simulation contained data from 2, 3, or 5 clinical trials, each containing a control (placebo or reference standard of care treatment) group and an experimental group.

We replicated this set of simulations with one important modification: we replaced the fixed effects model with a random effects model. Specifically, the fixed effects model prescribes *e* ~ *N*(*δ*, 1) with *δ*~ *N* (0.4, 0.13). The random effects model prescribes *e* ~ *N* (*δ*_*i*_, 1) with *δ*_*i*_ ~ *N*(*δ*, 0.1) and *δ* ~ *N* (0.4, 0.13), where *δ* indicates the population effect size for a given iteration and *δ*_*i*_ indicates the population effect size for a given trial *i* and an iteration. Results of these sensitivity analyses are qualitatively similar and may be found in the Additional file 1, available at https://osf.io/9yvd2/.

For each replication, we performed three types of analyses. Firstly, we conducted one-sided independent-samples *t*-tests for each of the trials. This resulted in a single *p*-value for each trial. Secondly, we calculated three kinds of meta-analytic 95% confidence intervals (CI) for the underlying effect size: a fixed effect method using inverse variance weighting, the DerSimonian and Laird (DSL) random effect method and the more conservative Hartung, Knapp, Sidik, and Jonkman (HKSJ) random effect method (see e.g., Appendix 1 in [19] for statistical details).

Thirdly, we conducted a one-sided independent-samples Bayesian *t*-test for the data of all trials combined. The vehicle of choice for quantifying the evidence was the one-sided Jeffreys Zellner-Siow (JZS) Bayes factor [20]. This one-sided Bayes factor quantifies the relative likelihood of the one-sided alternative hypothesis, the experimental group has a higher mean than the control group, against the null hypothesis, the experimental group has the same mean as the control group. The JZS Bayes factor is calculated by comparing the marginal likelihood of the data under the point null hypothesis to the marginal likelihood of the data under the alternative hypothesis, integrated over a range of plausible alternative hypotheses. The range of alternative hypotheses is given by a prior on the effect size parameter *δ*, which follows a Cauchy distribution centered on zero with a scale parameter of *r* = √2/2 (see [20], Equation in note 4). The Cauchy prior is typically referred to as an *objective prior*, because it satisfies a number of general desiderata [21, 22]. These include model selection consistency (data generated under a model should lead to a Bayes factor for this model converging to infinite as sample size increases) and predictive matching (there exists a minimum sample size for which one should not be able to distinguish between models, leading to a Bayes factor of 1). Contrary to a Normal prior, the Cauchy prior in addition satisfies the criterion of information consistency (different sequences of data with the same sample size for which likelihood ratios go to infinity should have corresponding Bayes factors that also go to infinity). We omit further details here for brevity but point the interested reader to [21].

Bayes factors were calculated using the BayesFactor R package [23]. For comparison, we calculated minimum Bayes factors [6, 24] using the pCalibrate package, also available in R [25]. Minimum Bayes factors quantify the upper bound of evidence against the null hypothesis for a number of priors under the alternative. As a result, it is more liberal than the JZS Bayes factor, leading to higher true and false positive rates. The reader interested in a comparison of the two Bayes factors is referred to the Additional file 1.

Once *p*-values, meta-analytic 95% CIs, and Bayes factors were obtained, different evidential thresholds and thresholds for clinical meaningfulness were combined to obtain true positive and false positive rates. For the null hypothesis significance test, the main manuscript reports a significance level *α* of 0.025, which corresponds to a two-sided test with significance level 0.05, followed by a check for the direction of the effect. Alternative significance levels *α* of 0.05 and 0.005 are reported in the Additional file 1. For the meta-analytic 95% CIs, the lower bound was used. For the Bayesian tests, Bayes Factor thresholds of 3, 10, and 50 were used. In order to decide whether a positive result counts as a true or false positive, a threshold needs to be established for an effect being clinically meaningful. We varied the clinically meaningful threshold from 0 (corresponding to a scenario where each non-zero effect is meaningful, no matter how small), 0.15, 0.3, and 0.45 (where only effect sizes higher than the respective numbers are clinically meaningful). There is a large literature on the clinically minimum important difference (CMID) in the biomedical literature and we used this as rough guidance for selecting these values. A simple approach is that a standardized effect of at least 0.5 is needed [26]. However, we erred on the side of being more lenient, since small effects may still be clinically meaningful, depending on the type of outcome, the setting, and the baseline risk involved [27, 28].

The quantification of true positives and false positives proceeds as follows. Taking as an example an *α* of 0.05 and a clinically meaningful threshold of 0.15, four scenarios are possible:

- *True positive*: The true effect size is higher than 0.15, and at least two trials produced a *p*-value lower than 0.05
- *False negative*: The true effect size is higher than 0.15, and fewer than two trials produced a *p*-value lower than 0.05
- *False positive*: The true effect size is lower than 0.15, and at least two trials produced a *p*-value lower than 0.05
- *True negative*: The true effect size is lower than 0.15, and fewer than two trials produced a *p*-value lower than 0.05

The false negative rate is equal to 1 minus the true positive rate and the true negative rate is equal to 1 minus the false positive rate. For the meta-analytic 95% CI, taking as an example a clinically meaningful threshold of 0.15, four scenarios are possible:

- *True positive*: The true effect size is higher than 0.15, and the lower bound of the meta-analytic 95% CI is higher than zero
- *False negative*: The true effect size is higher than 0.15, and the lower bound of the meta-analytic 95% CI is lower than zero
- *False positive*: The true effect size is lower than 0.15, and the lower bound of the meta-analytic 95% CI is higher than zero
- *True negative*: The true effect size is lower than 0.15, and the lower bound of the meta-analytic 95% CI is lower than zero

For the Bayesian tests, taking as an example a Bayes Factor threshold of 10 and a clinically meaningful threshold of 0.15, four scenarios are possible:

- *True positive*: The true effect size is higher than 0.15, and the Bayes Factor is higher than 10
- *False negative*: The true effect size is higher than 0.15, and the Bayes Factor is lower than 10
- *False positive*: The true effect size is lower than 0.15, and the Bayes Factor is higher than 10
- *True negative*: The true effect size is lower than 0.15, and the Bayes Factor is lower than 10

The code for these simulations is freely available on https://osf.io/9yvd2/.

## Results

**Fig. 1:**
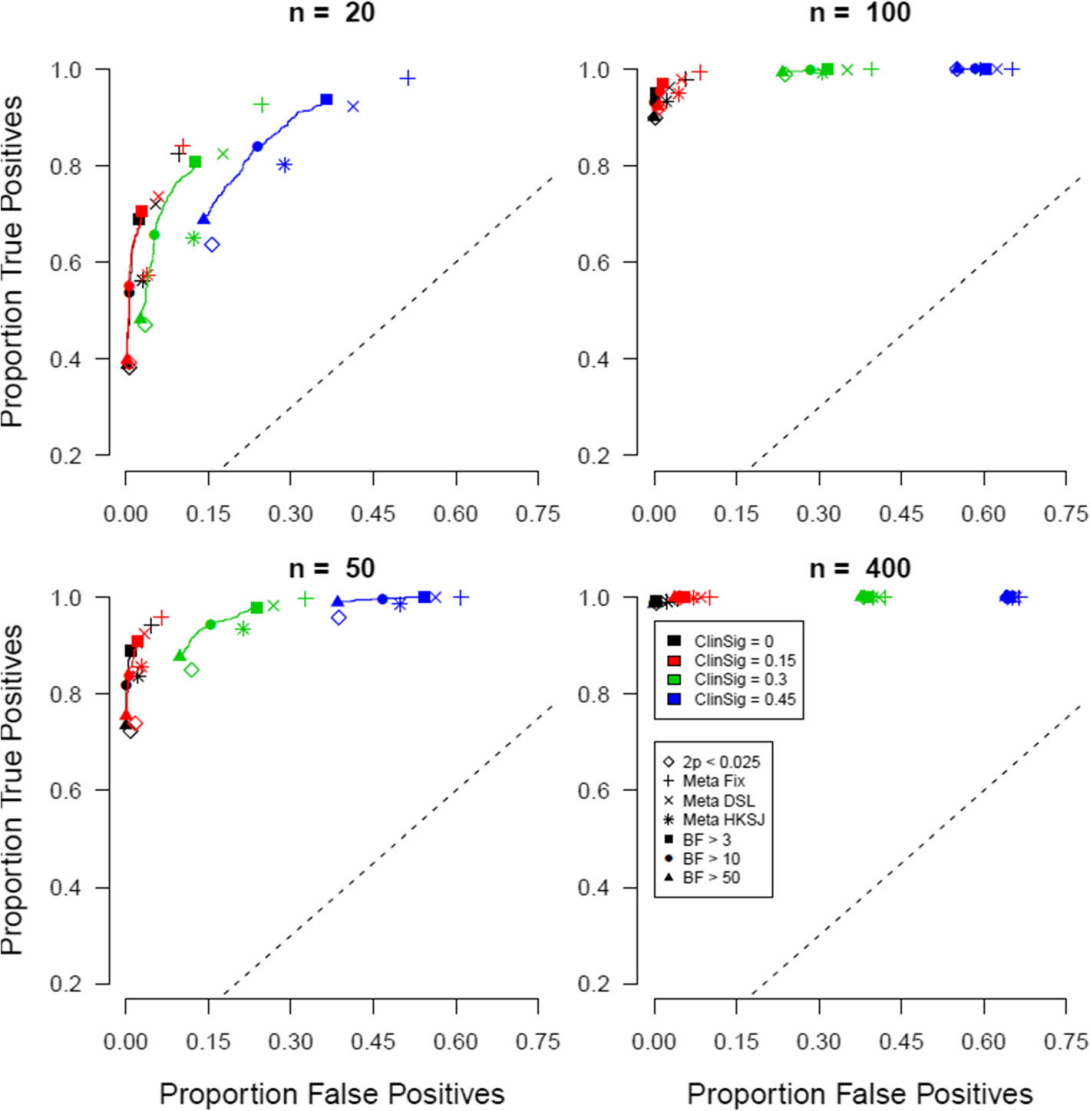
Proportion of true positives plotted against proportion of false positives when the prevalence of true null effects is 25%. Open symbols indicate conventional significance level *α*, closed symbols indicate Bayes factor thresholds, the +, x, and * symbols indicate meta-analytic 95% CIs, and different colors indicate different levels of clinically meaningful differences

For the simulation scenario of five clinical trials, true positives are plotted against false positives for different evidence criteria and for different thresholds for a clinically meaningful effect when the prevalence of the true null effect is 25% in Fig. 1. In all panels, the y-axis represents the proportion of true positives and the x-axis represents the proportion of false positives. Different panels indicate different numbers of participants per trial, and different colors represent different thresholds of a clinical meaningful effect. The ideal scenario of detecting 100% true positives and 0% false positives is represented by the top-left of each panel. Note that for the conventional statistical significance results and a clinically meaningful threshold of 0, false positive rates can be analytically calculated with 1-((1-*α*)^5 + (1-*α*)^4*α*5). The expression for the false positive rates is less straightforward when the effect size of 0 tested in null hypothesis significance testing differs from the clinically meaningful effect value used to categorize the underlying population effect as true or false. To keep results consistent, we display simulation results for all levels of clinical meaningful effects.

The results show to what extent working with more stringent criteria for statistical evidence reduces the proportion of false positives at the expense of reducing the proportion of true positives. This is as it should be, more conservative decision criteria trade off a reduction in the endorsement of ineffective medication against a lower endorsement of effective medication.

When looking at the performance of the meta-analytic 95% CI, we see that across the board the meta-analytic methods are associated with a higher true positive and a higher false positive rate. Regardless of sample size and clinical significance threshold, the HKSJ method is more conservative than the DSL method, which is in turn more conservative than the fixed effects method.

Next, we compare the results of the conventional statistical significance results to Bayesian testing results. Across the board, the panels show that Bayesian criteria for decision making are slightly more to the top and more to the left of conventional decision criteria in particular when small sample sizes are involved. For instance, for *n* = 20, compared to two trials with *p* < 0.025, a decision rule based on an overall Bayes factor higher than 50 results in a slightly higher proportion of true positives *and* a slightly lower proportion of false positives. Using more stringent or more relaxed criteria for statistical evidence shifts the balance between proportions true and false positives, but does not change the fact that for every two-significant-trial criterion, there is a corresponding BF > x criterion with more true positives and fewer false positives combined (see Additional file 1 for details). The BF criteria seem better than both the conventional criteria and the meta-analytic methods in particular with large meaningful clinical difference thresholds. The difference between the two inferential methods becomes smaller with larger trials and is not discernible with 400 participants per arm.

**Fig. 2:**
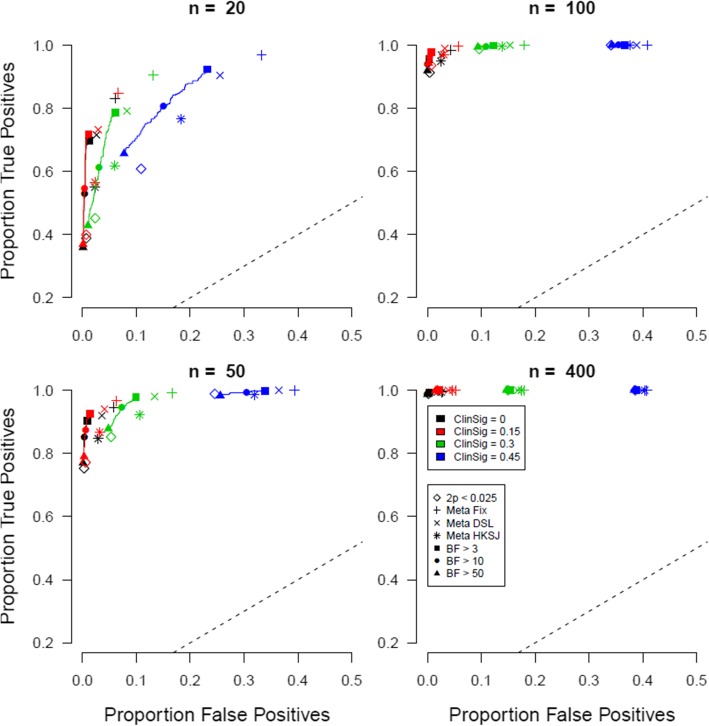
Proportion of true positives plotted against proportion of false positives when the prevalence of true null effects is 50%. Open symbols indicate conventional significance level *α*, closed symbols indicate Bayes factor thresholds, the +, x, and * symbols indicate meta-analytic 95% CIs, and different colors indicate different levels of clinically meaningful differences

To what extent do the results differ when we assume a higher prevalence of true null effects? The answer for a null-effect prevalence of 50% can be found in Fig. 2. The layout is similar to that of Fig. 1.

Comparing the results for a 50% prevalence of true null effects to those for a 25% prevalence of true null effects, one clear observation is that the proportion of false positives dwindles across the board. The majority of false positives are caused by effects that come from the non-null normal distribution, but do not pass the clinically meaningful threshold. This means that the more true negatives there are, the lower the proportion of false positives is. The proportion of true positives is unaffected, which is again as it should be, given that nothing changed across these simulations within the distribution of non-null effects.

Importantly, we again find the pattern that Bayesian criteria are typically associated with a combination of higher true positive rates and lower false positive rates than conventional statistical significance-based decision criteria, especially with small sample sizes (*n* = 20 per arm) and somehow more prominently with larger clinically meaningful differences. We also replicate the result that the Bayesian methods perform slightly better than both the *p*-value criterion and the meta-analytic CIs when employed for decision making purposes.

**Fig. 3:**
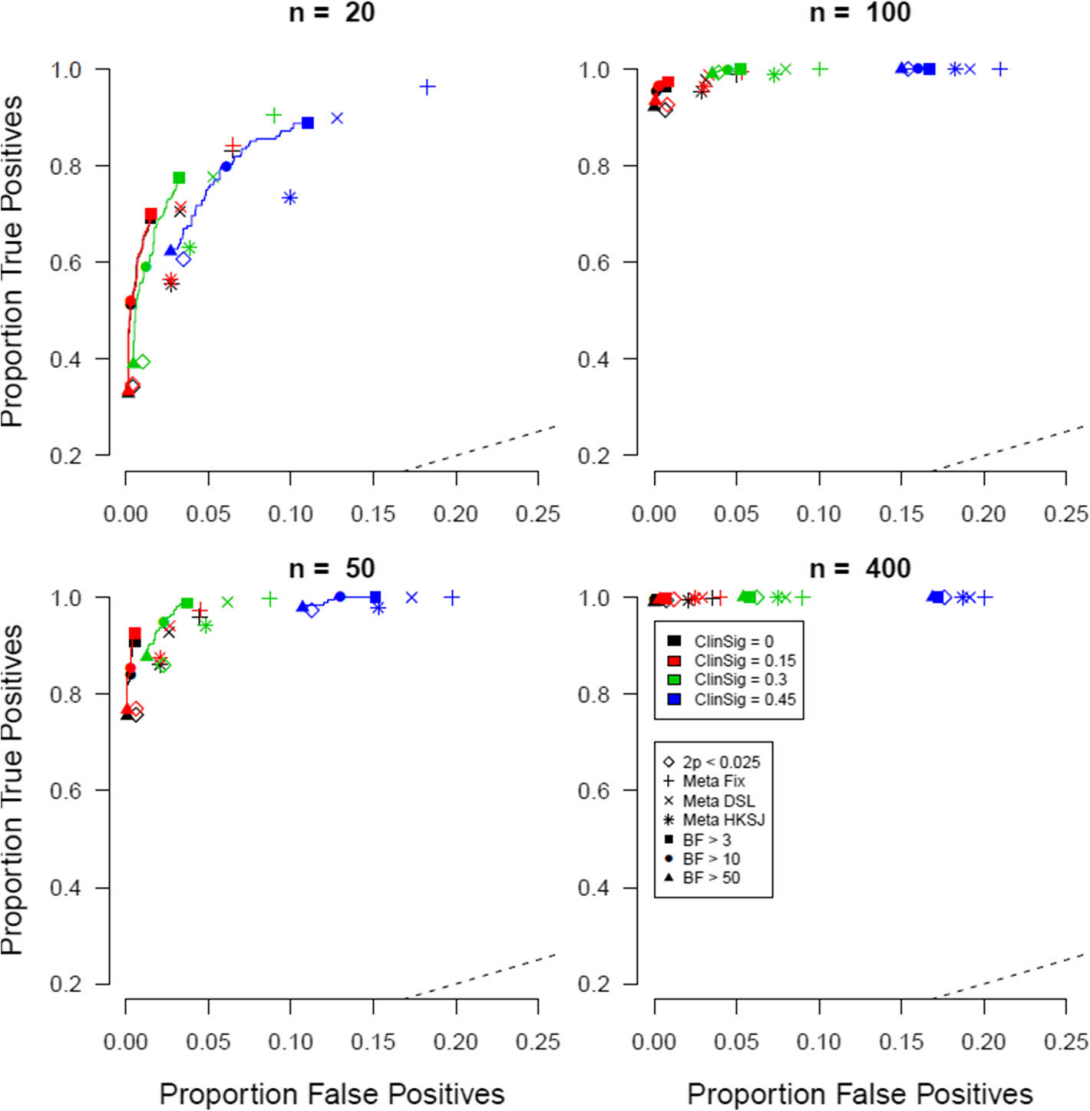
Proportion of true positives plotted against proportion of false positives when the prevalence of true null effects is 75%. Open symbols indicate conventional significance level *α*, closed symbols indicate Bayes factor thresholds, the +, x, and * symbols indicate meta-analytic 95% CIs, and different colors indicate different levels of clinically meaningful differences

Results for a null-effect prevalence of 75% can be found in Fig. 3. The layout is similar to that of Fig. 2.

Consistent with the comparison of previous results, we see the reduction in false positives across the board. The better performance of the Bayesian evaluation criterion, both in terms of true positives and in terms of false positives is seen again with small sample sizes.

**Fig. 4:**
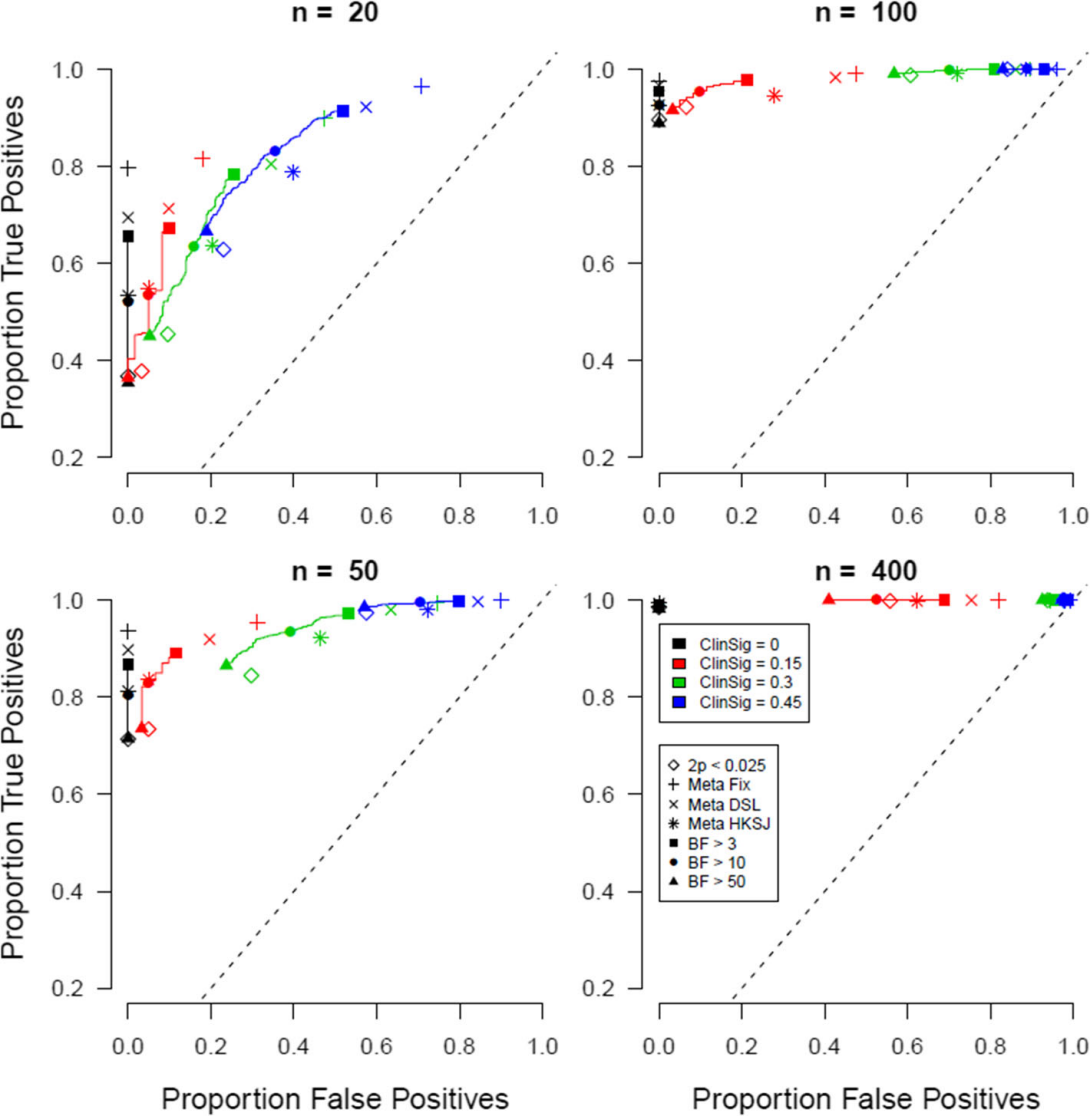
Proportion of true positives plotted against proportion of false positives when the prevalence of true null effects is 0%. Open symbols indicate conventional significance level *α*, closed symbols indicate Bayes factor thresholds, the +, x, and * symbols indicate meta-analytic 95% CIs, and different colors indicate different levels of clinically meaningful differences

Lastly, results are plotted for an ideal scenario in which there exist only non-null effects, or a prevalence of true null effects of 0% (all tested medications are truly effective). Results can be found in Fig. 4.

The scenario of no null effects displays the previous pattern of results in its most extreme: identical true positive rates, relatively bad false positive rates when the clinically meaningful threshold is anything higher than zero, and better performance of the Bayesian evaluation criterion.

Results for the simulation scenarios of three clinical trials were qualitatively similar to those for five clinical trials reported here. For two clinical trials, the significance method (i.e., both trials were significant) performed very similar to the Bayesian method. Detailed results can be found in the Additional file 1.

In order to get a better handle on the performance of the different types of meta-analysis, we examined for different sample sizes how often the meta-analytic confidence intervals included the true population effect size. The results are plotted in Table 1.

**Table 1:** Proportion of times the true population effect size was included in the three different meta-analytic confidence intervals. Rows represent the three meta-analytic methods, columns represent different sample sizes

	20	50	100	400
Fixed	.856	.887	.893	.903
DSL	.928	.933	.936	.938
HKSJ	.944	.946	.950	.948

Inspection of the table shows that the coverage of the HKSJ method (the probability that the true effect is included in the 95% confidence interval) was better than the coverage of the DSL method which was in turn better than the coverage of the fixed effects method. HKSJ is well-known to be a better method in most circumstances and this is also consistent with what we observed.

## Discussion

In this study, we simulated clinical trial data comparing an experimental group to a control group (placebo or other standard of care). Simulations differed in the number of clinical trials conducted, the proportion of true null effects versus non-null effects, and the number of participants in each trial. For all simulations, we compared the true and false positive rates when strength of evidence was quantified by counting the number of significant results, quantified by *p*-values lower than a certain significance level *α*, by constructing meta-analytic 95% confidence intervals and assessing their overlap with a clinically meaningful effect, or by Bayes factor assessing the overall strength of evidence provided by the five trials. True and false positive rates were defined using four different thresholds of an effect being considered clinically meaningful.

The take-home message when surveying the results of all simulations together is that quantifying evidence with Bayes factors compared to counting the number of statistically significant results or meta-analytic confidence intervals leads to a higher true positive rate for a given false positive rate, or a lower false positive rate for a given true positive rate, especially when relatively small trials are involved. We find this result irrespective of the significance level or Bayes factor threshold chosen. Our results get more pronounced as the incidence rate of true null effects gets lower. The latter should be particularly relevant in the assessment of stage III clinical trials, where the prevalence of truly null effects is arguably lower than for stage I and II trials.

Meta-analysis is a powerful tool for estimating the size of an unknown population effect size across the results of several independent studies. In our simulations, we confirmed that the meta-analytic confidence intervals often captured the true population effect size, and HKSJ performed better than DSL random effects which in turn performed better than fixed effects. For purposes of making a dichotomous yes/no decision however, Bayesian methods appeared to be the (slightly) superior choice in scenarios where sample size was low. Apparently, the strength of meta-analytic methods is in making assessments about the continuum of the effect size and its uncertainty, rather than binary decisions.

Bayesian methods for statistical inference have been recommended for use in the analysis of clinical trials before [29, 30], and have been discussed in the context of the regulatory process ([31], see also the rest of this special issue), but they are still much less commonly used than *p*-values from null hypothesis significance testing. In fact, across the biomedical literature, null hypothesis significance testing seems to be about 100-fold more commonly used than Bayesian inference [32]. While previously underuse of Bayesian methods may have been excused by the unavailability of means to carry out these analyses, recently a number of free and user-friendly types of software have been developed [23, 33, 34], such that technical limitations should no longer be a concern.

Some caveats need to be discussed. It is important to stress that the approval process does not hinge exclusively on an evaluation of the available statistical evidence in a strictly quantitative manner, nor do we wish for quantitative automation to replace nuanced reasoning. Rather, qualitative aspects of the appropriateness of the design, conduct and analysis of the trial should continue to be evaluated as well as the relevance of the outcomes used, potential safety implications, and side-effects and harms that could affect the benefit-risk ratio. Each of these considerations will factor into the endorsement process and can lead to a different desired tradeoff for maximizing true positives versus minimizing false positives. Our simulations should provide some guidance on how this tradeoff should be influenced by the anticipated distribution of effects in the relevant drug group or subfield. Our results and subsequent recommendations should be taken to apply to the statistical component of the evaluation process, and should be combined with other utilities to come to the appropriate analysis strategy.

We should also caution that the absolute magnitude of difference in performance between the Bayesian and *p*-value-based criteria was often small or negligible in several of the simulations. Moreover, it is typically difficult to know what the likely values are for the frequency of null effects in specific fields and applications. The advantage of the Bayesian criteria seemed clearer when sample sizes were small and clinically meaningful differences were not small. With large sample sizes and small clinically meaningful differences, the three inferential approaches seemed to have very similar performance.

Finally, we used popular but arbitrary cut-offs for both statistical significance and Bayes factors and in fact both of these take continuous values. Such dichotomization may cause loss of information for both types of criteria.

The modest superiority of the Bayesian approach may be due to the fact that it considers all evidence in a cumulative manner, while the rule of having two statistically significant results adds a further dichotomization in counting “positive” and “negative” trials, with further loss of information.

## Conclusion

Allowing for these caveats, evaluating the results of multiple clinical trials with Bayes factors is a useful approach that, in some circumstances may lead to a higher true positive rate for the same false positive rate, compared to an analysis strategy based on simply counting the number of significant results. Decisions on the appropriate Bayes factor threshold should be based on additional utilities that are not part of the statistical process and may also be informed by the anticipated prevalence of true null effects.

## Supplementary information

**Additional file 1.** True and false positive rates for different criteria of evaluating statistical evidence from clinical trials.

## Data Availability

Code for reproducing the analyses reported in the manuscript may be obtained from https://osf.io/9yvd2/.

## Abbreviations

CI: Confidence intervals
CMID: Clinically minimum important difference
DSL: DerSimonian and Laird random effect method
FDA: US Food and Drug Administration
HKSJ: Hartung, Knapp, Sidik, and Jonkman random effect method
JZS: Jeffreys Zellner-Siow
RCT: Registered clinical trial
