# Supplementary material for: True and false positive rates for different criteria of evaluating statistical evidence from clinical trials
Source: BMC Med Res Methodol. 2019 Nov 27;19:218. doi: 10.1186/s12874-019-0865-y (PMC6882054; doi:10.1186/s12874-019-0865-y)
Supplement: Supplementary file 1 — Additional file 1. True and false positive rates for different criteria of evaluating statistical evidence from clinical trials. [file 12874_2019_865_MOESM1_ESM.docx]

**SUPPLEMENTARY MATERIAL**

True and False Positive Rates for Different Criteria of Evaluating Statistical Evidence from Clinical Trials

Don van Ravenzwaaij^1^ and John P. A. Ioannidis^2^

^1^Department of Psychology, University of Groningen, ^2^Departments of Medicine, of Health Research and Policy, and of Statistics and Meta-Research Innovation Center at Stanford (METRICS), Stanford University

Correspondence concerning this article should be addressed to:

Don van Ravenzwaaij

University of Groningen, Department of Psychology

Grote Kruisstraat 2/1, Heymans Building, room 169

9712 TS Groningen, The Netherlands

Ph: (+31) 50 363 7021

E–mail should be sent to d.van.ravenzwaaij@rug.nl.

This document contains supplementary material to “True and False Positive Rates for Different Criteria of Evaluating Statistical Evidence from Clinical Trials” [1]. The first part of this document contains the four plots from the main manuscript, now with (1) significance level *α* 0.05 and 0.0005; and (2) minimum Bayes factors added.

The second part of this document contains four plots for the simulation scenario where the number of clinical trials was 3, instead of the 5 reported in the main manuscript.

The third part of this document contains four plots for the simulation scenario where the number of clinical trials was 2, instead of the 5 reported in the main manuscript.

The last part of this document contains simulations for which the fixed effects model was replaced with a random effects model. Specifically, the fixed effects model prescribes *e* ~ *N*(*δ*, 1) with *δ*~ *N*(0.4, 0.13), where *δ* indicates the population effect size for a given iteration. The random effects model prescribes *e* ~ *N*(*δ_i_*, 1) with *δ_i_* ~ *N*(*δ*, 0.1) and *δ* ~ *N*(0.4, 0.13), where *δ* indicates the population effect size for a given iteration and *δ_i_* indicates the population effect size for a given trial *i* and an iteration. For details about the general simulation set-up, we refer to the main manuscript.

Our results are summarized in the same way as in the main manuscript, which results in 4 individual figures.

Part I

True positives are plotted against false positives for different significance levels *α*, Bayes factor thresholds (both JZS and minimum Bayes factors), and thresholds for a clinically meaningful effect when the prevalence of the true null effect is 25%, 50%, 75%, and 0% in Figures S1, S2, S3, and S4 respectively. In all panels, the y-axis represents the proportion of true positives and the x-axis represents the proportion of false positives. Different panels indicate different numbers of participants per trial, and different colors represent different thresholds of a clinical meaningful effect.

Fig S1 about here

**Fig S1***.* Proportion of true positives plotted against proportion of false positives when the prevalence of true null effects is 25%. See figure legend for details.

Fig S2 about here

**Fig S2**. Proportion of true positives plotted against proportion of false positives when the prevalence of true null effects is 50%. See figure legend for details.

Fig S3 about here

**Fig S3**. Proportion of true positives plotted against proportion of false positives when the prevalence of true null effects is 75%. See figure legend for details.

Fig S4 about here

**Fig S4**. Proportion of true positives plotted against proportion of false positives when the prevalence of true null effects is 0%. See figure legend for details.

Comparing these results to those obtained in Figures 1 through 4 of the main manuscript shows that the minimum Bayes factor is more liberal than the JZS Bayes factor, but both methods fall along the same curve in the ROC plot. The different significance levels *α* seem to fall along the same curve as the meta-analytic methods*.*

Results for a null-effect prevalence of 50% can be found in Fig S2. The layout is similar to that of Fig S1.

Part II

This part discussion results for the simulation scenario where the total number of clinical trials was 3. True positives are plotted against false positives for different significance levels *α*, Bayes factor thresholds (both JZS and minimum Bayes factors), and thresholds for a clinically meaningful effect when the prevalence of the true null effect is 25%, 50%, 75%, and 0% in Figures S5, S6, S7, and S8 respectively. The layout is similar to that of Figures S1 through S4.

Fig S5 about here

**Fig S5***.* Proportion of true positives plotted against proportion of false positives when the prevalence of true null effects is 25%. See figure legend for details.

Fig S6 about here

**Fig S6**. Proportion of true positives plotted against proportion of false positives when the prevalence of true null effects is 50%. See figure legend for details.

Fig S7 about here

**Fig S7**. Proportion of true positives plotted against proportion of false positives when the prevalence of true null effects is 75%. See figure legend for details.

Fig S8 about here

**Fig S8**. Proportion of true positives plotted against proportion of false positives when the prevalence of true null effects is 0%. See figure legend for details.

Comparing these results to those obtained for five clinical trials, we find a qualitatively similar pattern: the Bayesian methods perform better than the other methods. A direct comparison between the results for the JZS Bayes factor with decision threshold 50 and the two-significant trial criterion with significance level 0.025 shows that in many cases the Bayes factor simultaneously has a higher proportion of true positives and a lower proportion of false positives.

Part III

This part discussion results for the simulation scenario where the total number of clinical trials was 2. True positives are plotted against false positives for different significance levels *α*, Bayes factor thresholds (both JZS and minimum Bayes factors), and thresholds for a clinically meaningful effect when the prevalence of the true null effect is 25%, 50%, 75%, and 0% in Figures S9, S10, S11, and S12 respectively. The layout is similar to that of Figures S1 through S4.

Fig S9 about here

**Fig S9***.* Proportion of true positives plotted against proportion of false positives when the prevalence of true null effects is 25%. See figure legend for details.

Fig S10 about here

**Fig S10**. Proportion of true positives plotted against proportion of false positives when the prevalence of true null effects is 50%. See figure legend for details.

Fig S11 about here

**Fig S11**. Proportion of true positives plotted against proportion of false positives when the prevalence of true null effects is 75%. See figure legend for details.

Fig S12 about here

**Fig S12**. Proportion of true positives plotted against proportion of false positives when the prevalence of true null effects is 0%. See figure legend for details.

Comparing these results to those obtained for five and three clinical trials, we find that it is much harder to distinguish between the Bayesian methods and the two-significant trial criterion. This is perhaps not surprising: when all trials need to be significant, there is less flexibility then when only a subset needs to be.

Part IV

This part describes the simulation results for the random effects model. True positives are plotted against false positives for different significance levels *α*, Bayes factor thresholds, thresholds for a clinically meaningful effect, prevalence of the true null effect, and number of clinical trials in Fig S13 through Fig S24. The order of presentation of these figures is analogous to those for the fixed effects model that were presented in Fig S1 through S12. All results for the random effects model are qualitatively similar to those obtained for the fixed effects model.

Fig S13 about here

**Fig S13***.* Proportion of true positives plotted against proportion of false positives when the prevalence of true null effects is 25% for five clinical trials. See figure legend for details.

Fig S14 about here

**Fig S14***.* Proportion of true positives plotted against proportion of false positives when the prevalence of true null effects is 50% for five clinical trials. See figure legend for details.

Fig S15 about here

**Fig S15***.* Proportion of true positives plotted against proportion of false positives when the prevalence of true null effects is 75% for five clinical trials. See figure legend for details.

Fig S16 about here

**Fig S16***.* Proportion of true positives plotted against proportion of false positives when the prevalence of true null effects is 0% for five clinical trials. See figure legend for details.

Fig S17 about here

**Fig S17***.* Proportion of true positives plotted against proportion of false positives when the prevalence of true null effects is 25% for three clinical trials. See figure legend for details.

Fig S18 about here

**Fig S18***.* Proportion of true positives plotted against proportion of false positives when the prevalence of true null effects is 50% for three clinical trials. See figure legend for details.

Fig S19 about here

**Fig S19***.* Proportion of true positives plotted against proportion of false positives when the prevalence of true null effects is 75% for three clinical trials. See figure legend for details.

Fig S20 about here

**Fig S20***.* Proportion of true positives plotted against proportion of false positives when the prevalence of true null effects is 0% for three clinical trials. See figure legend for details.

Fig S21 about here

**Fig S21***.* Proportion of true positives plotted against proportion of false positives when the prevalence of true null effects is 25% for two clinical trials. See figure legend for details.

Fig S22 about here

**Fig S22***.* Proportion of true positives plotted against proportion of false positives when the prevalence of true null effects is 50% for two clinical trials. See figure legend for details.

Fig S23 about here

**Fig S23***.* Proportion of true positives plotted against proportion of false positives when the prevalence of true null effects is 75% for two clinical trials. See figure legend for details.

Fig S24 about here

**Fig S24***.* Proportion of true positives plotted against proportion of false positives when the prevalence of true null effects is 0% for two clinical trials. See figure legend for details.

In order to get a better handle on the performance of the different types of meta-analysis, we examined for different sample sizes how often the meta-analytic confidence intervals included the true population effect size. The results are plotted in Table 1.

Table S1 about here

Inspection of the table shows that the coverage of the HKSJ method (the probability that the true effect is included in the 95% confidence interval) was better than the coverage of the DSL method which was in turn better than the coverage of the fixed effects method.

Overall, our results are very much in line with those reported in [1].

References

[1] van Ravenzwaaij D, Ioannidis JPA. True and False Positive Rates for Different Criteria of Evaluating Statistical Evidence from Clinical Trials. Manuscript submitted for publication.

Table S1. Proportion of times the true population effect size was included in the three different meta-analytic confidence intervals. Rows represent the three meta-analytic methods, columns represent different sample sizes.

|  | 20 | 50 | 100 | 400 |
| --- | --- | --- | --- | --- |
| Fixed | .839 | .850 | .826 | .670 |
| DSL | .922 | .924 | .915 | .887 |
| HKSJ | .947 | .947 | .951 | .948 |


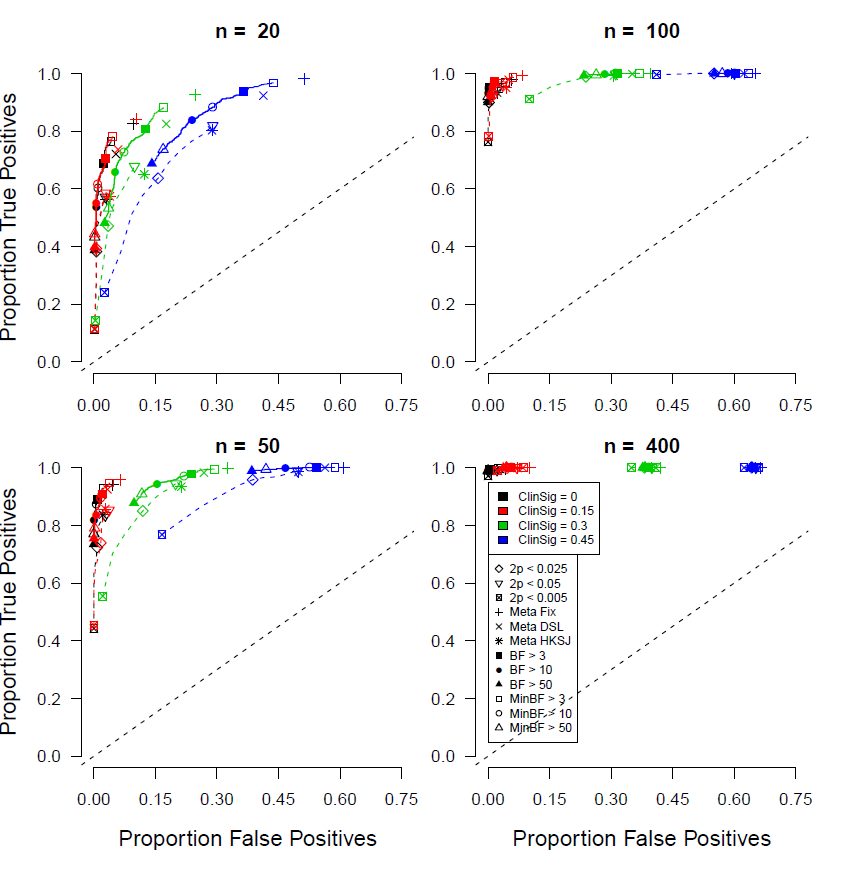


*Figure S1*


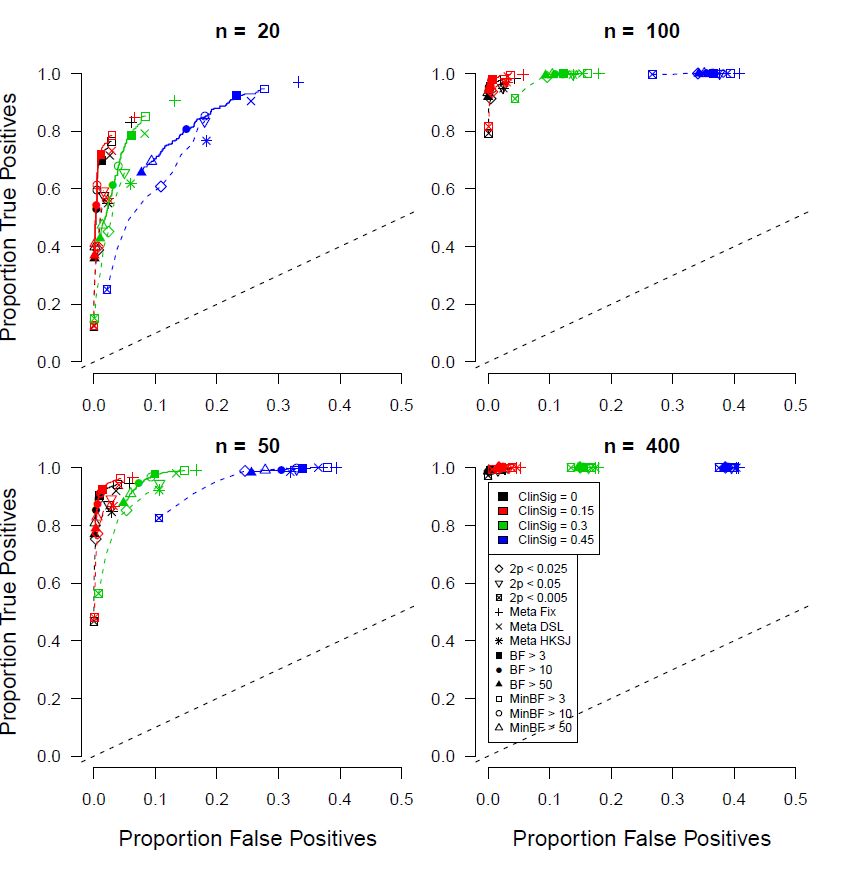


*Figure S2*


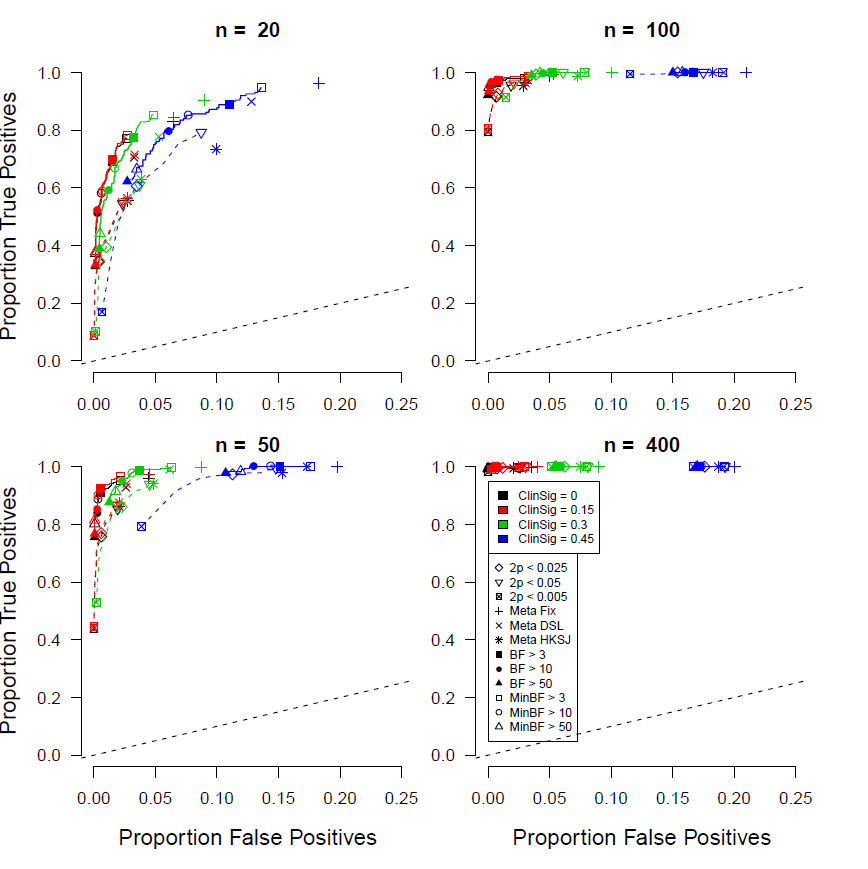


*Figure S3*


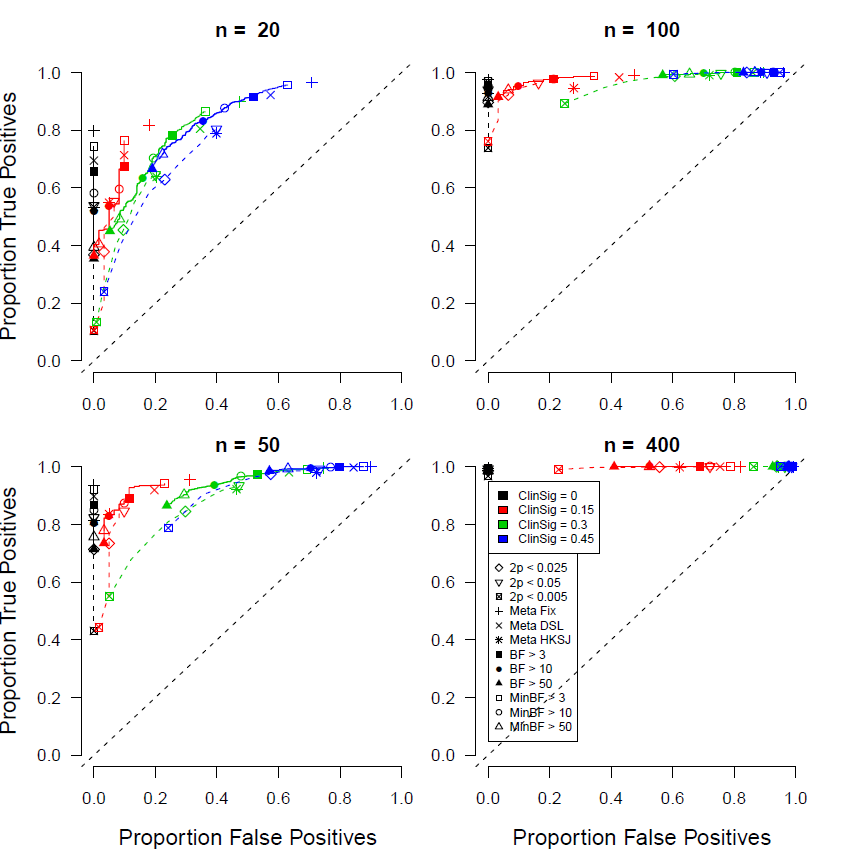


*Figure S4*


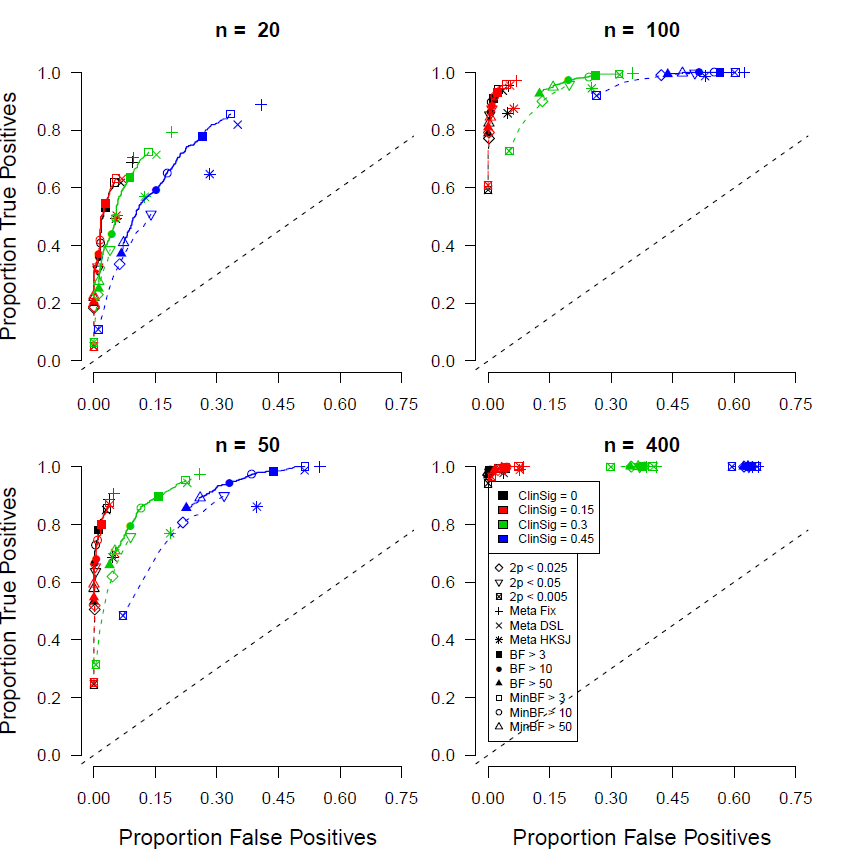


*Figure S5*


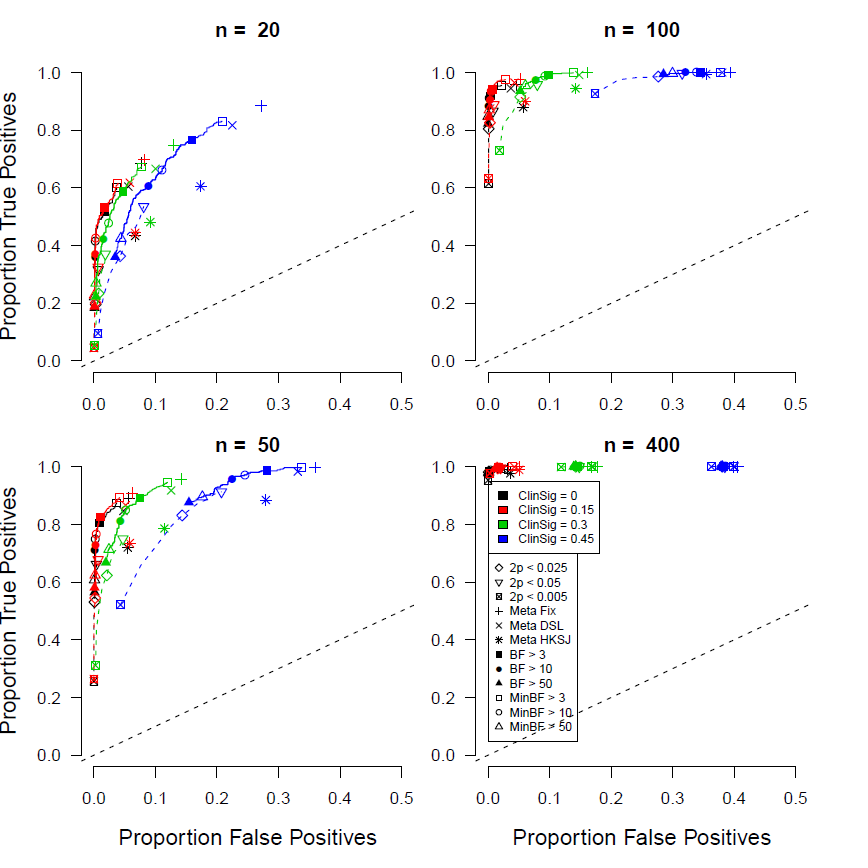


*Figure S6*


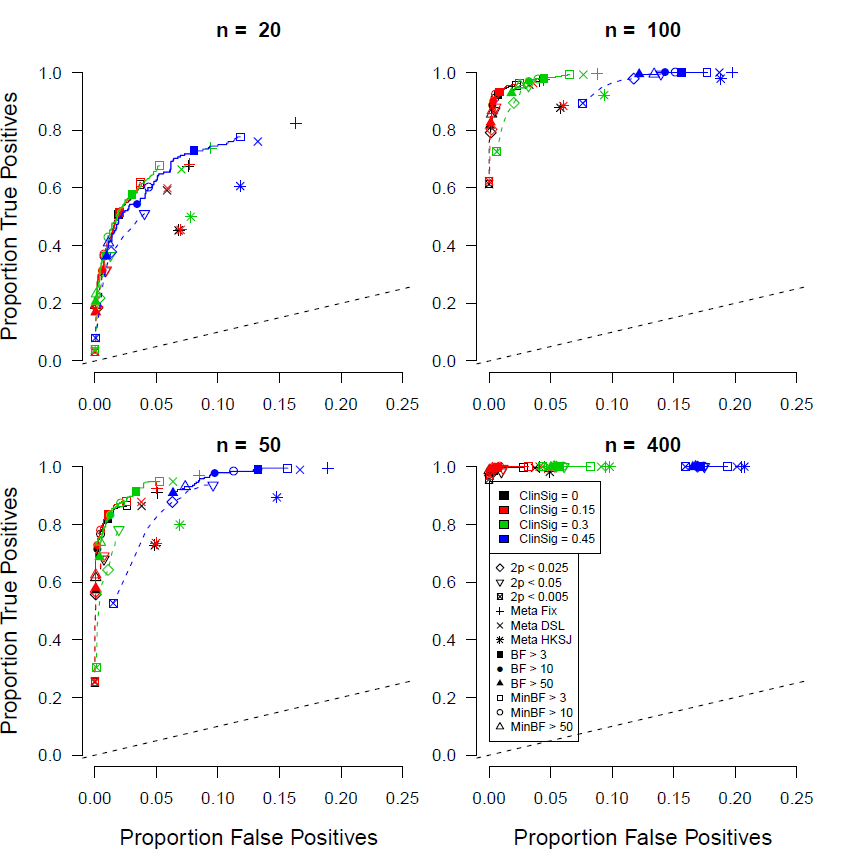


*Figure S7*


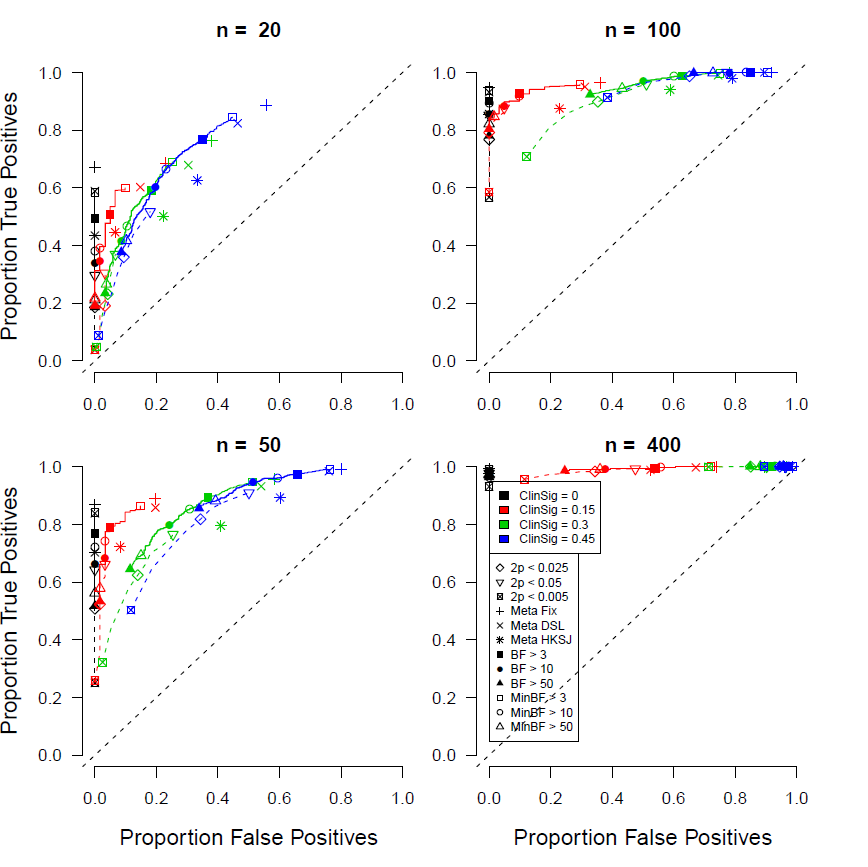


*Figure S8*


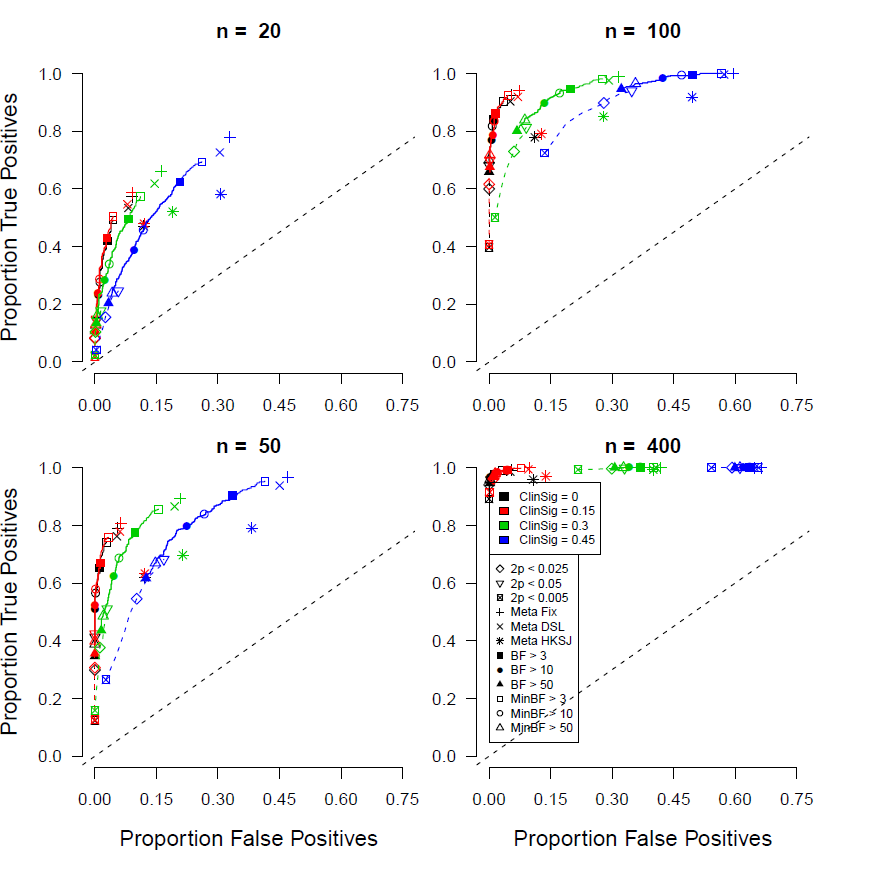


*Figure S9*


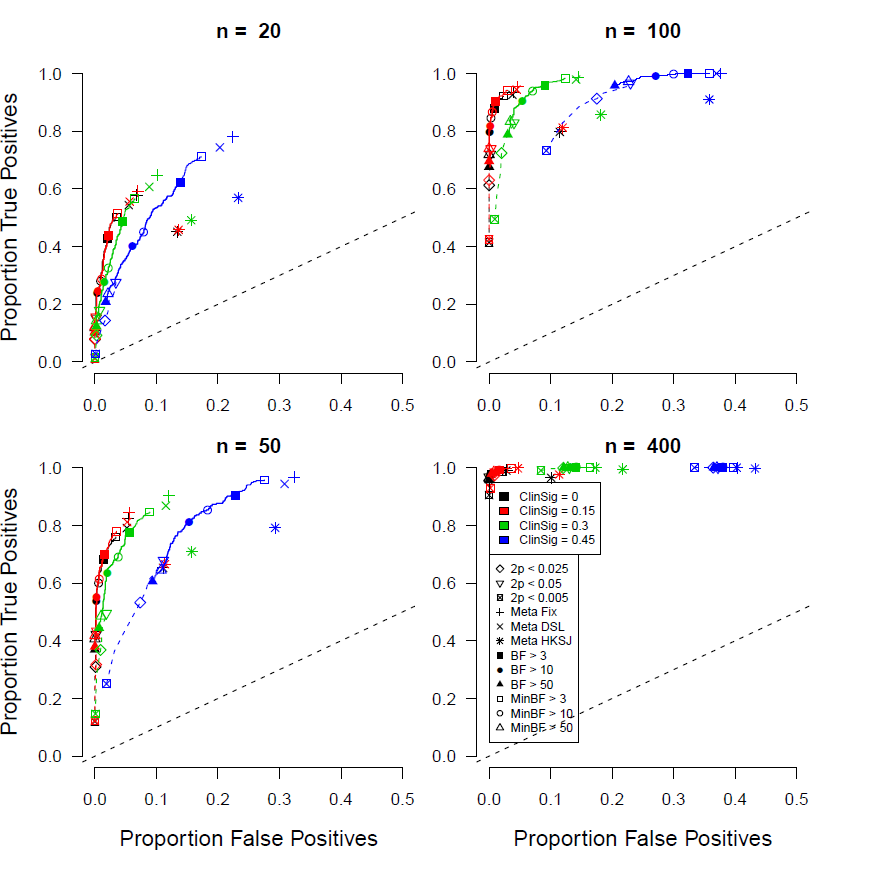


*Figure S10*


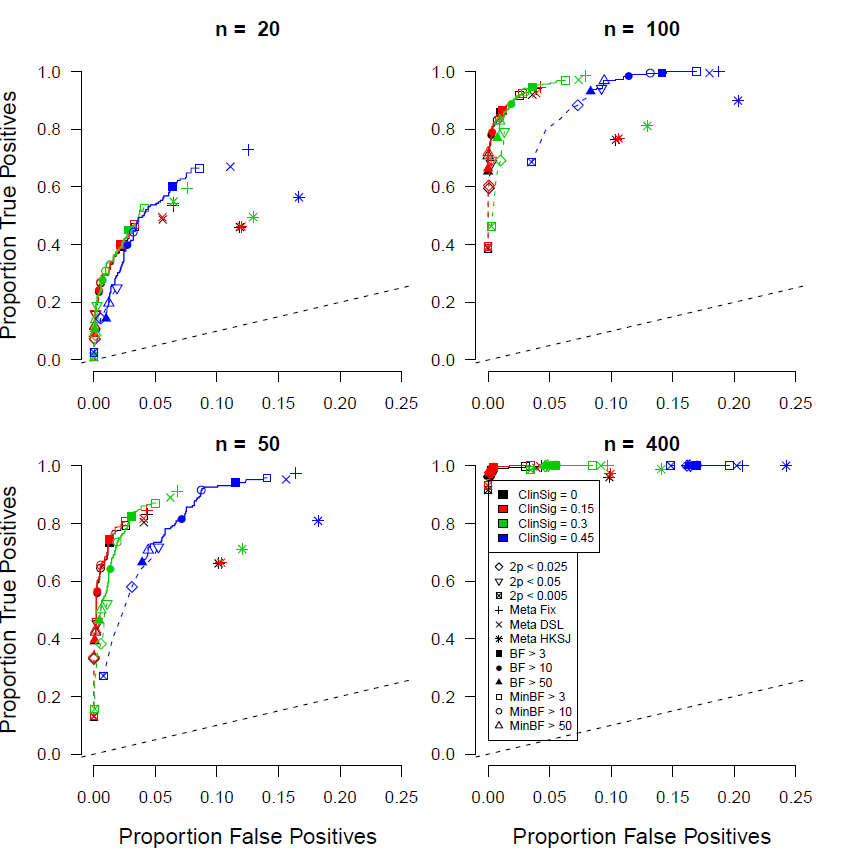


*Figure S11*


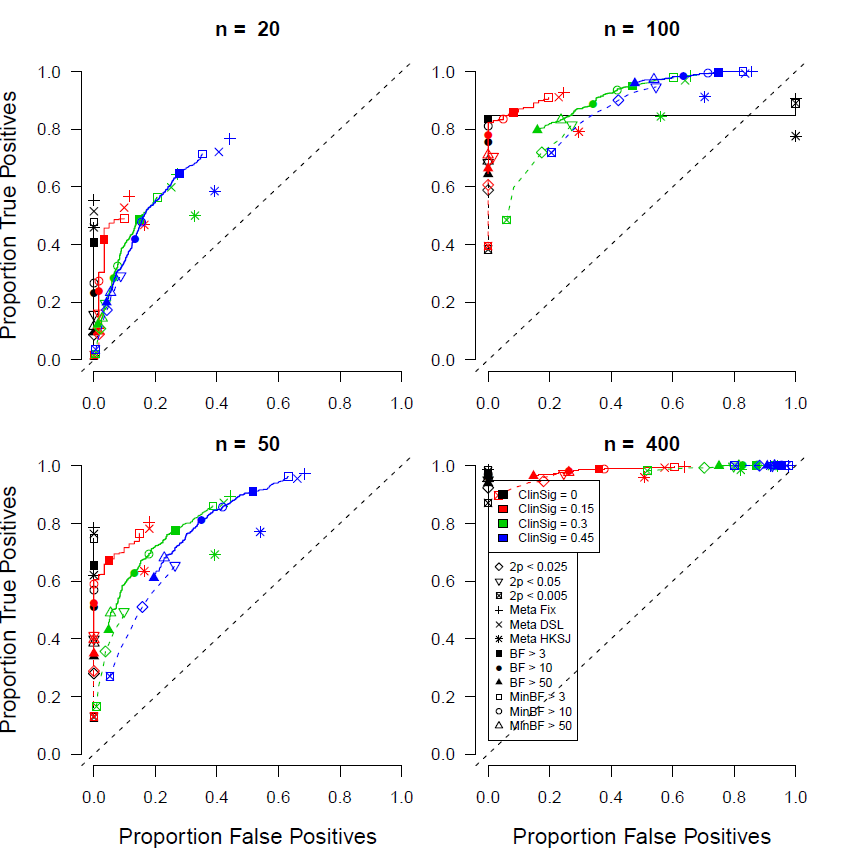


*Figure S12*

*
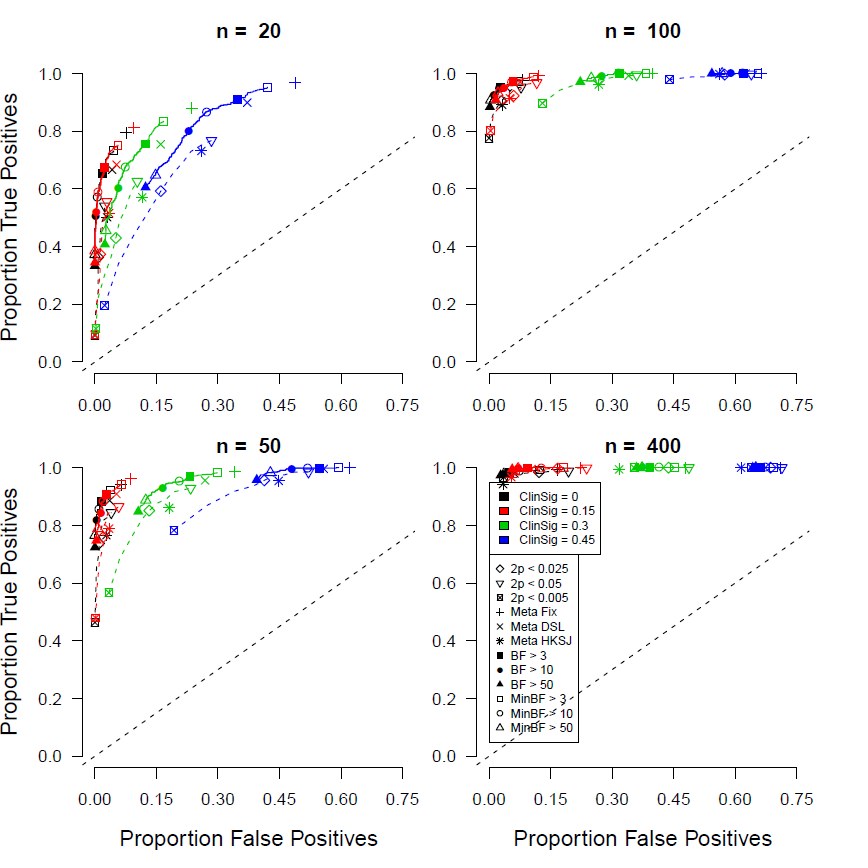
*

*Figure S13*

*
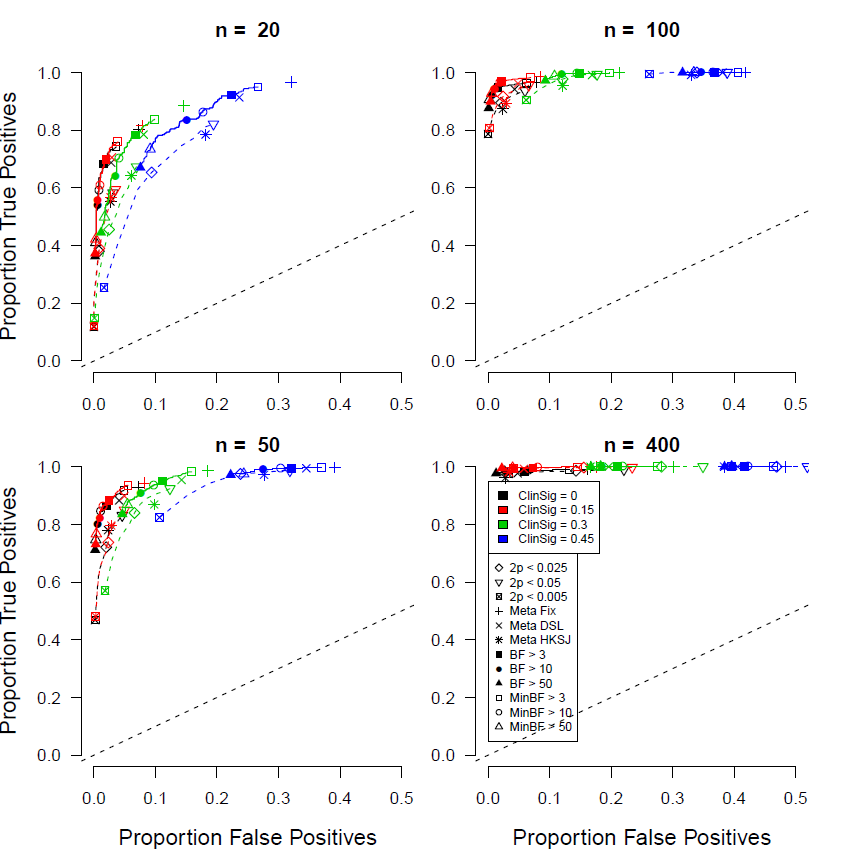
*

*Figure S14*


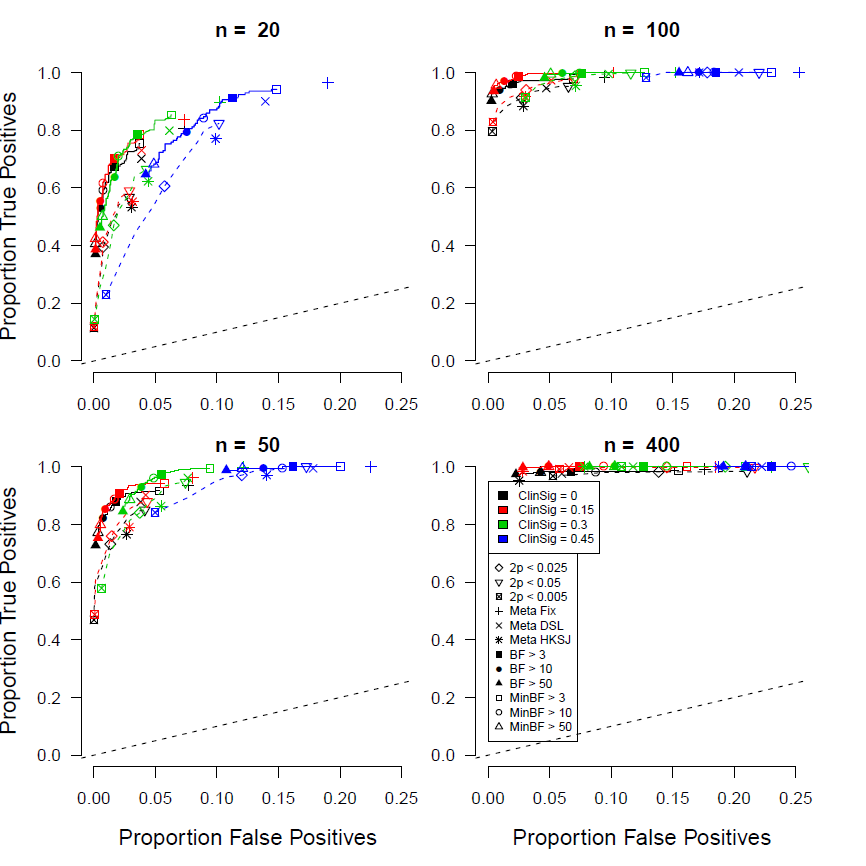


*Figure S15*


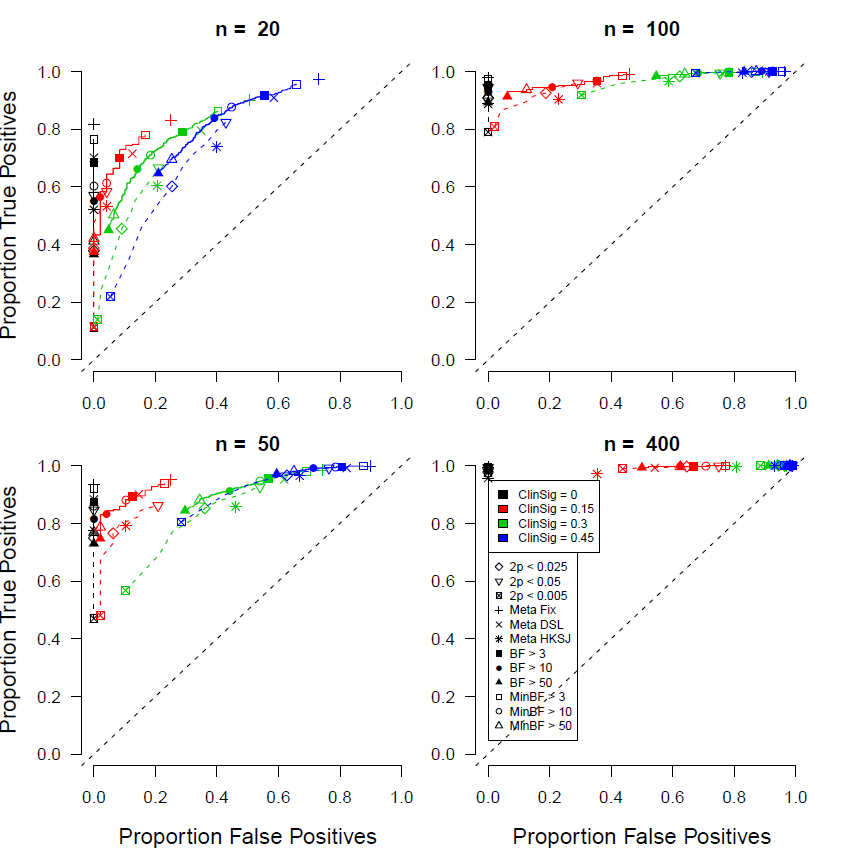


*Figure S16*

*
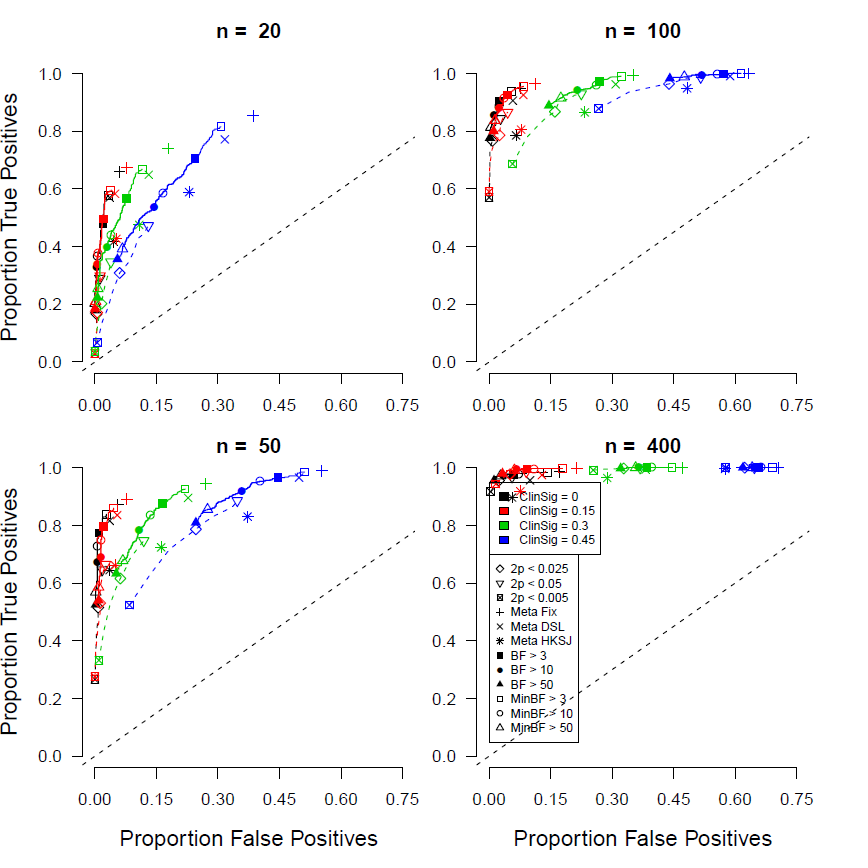
*

*Figure S17*

*
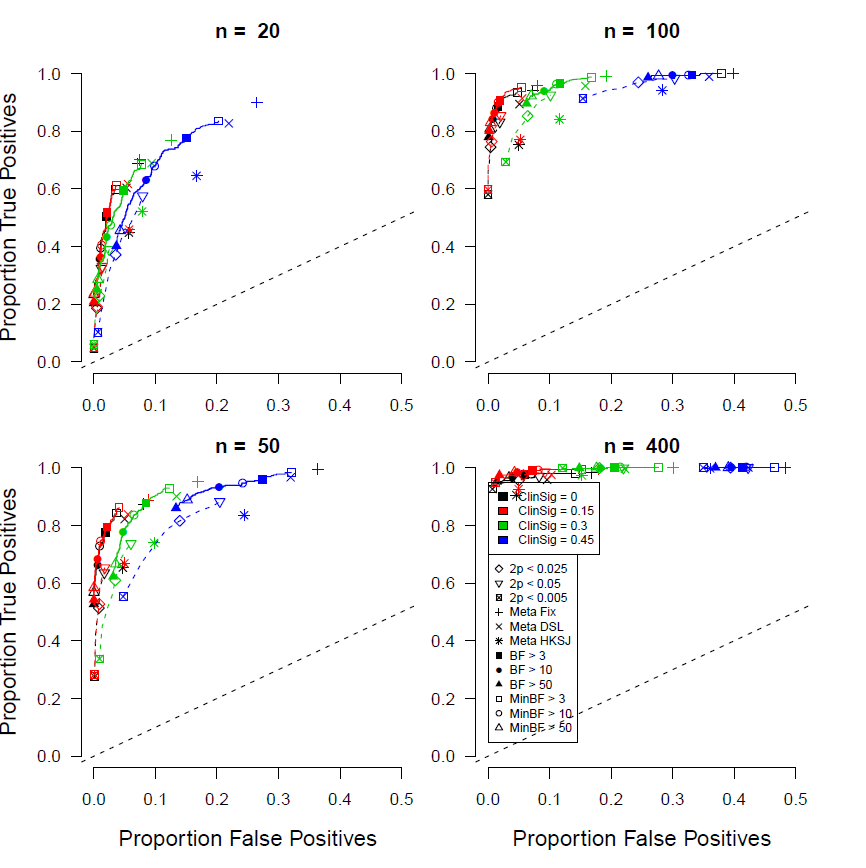
*

*Figure S18*

*
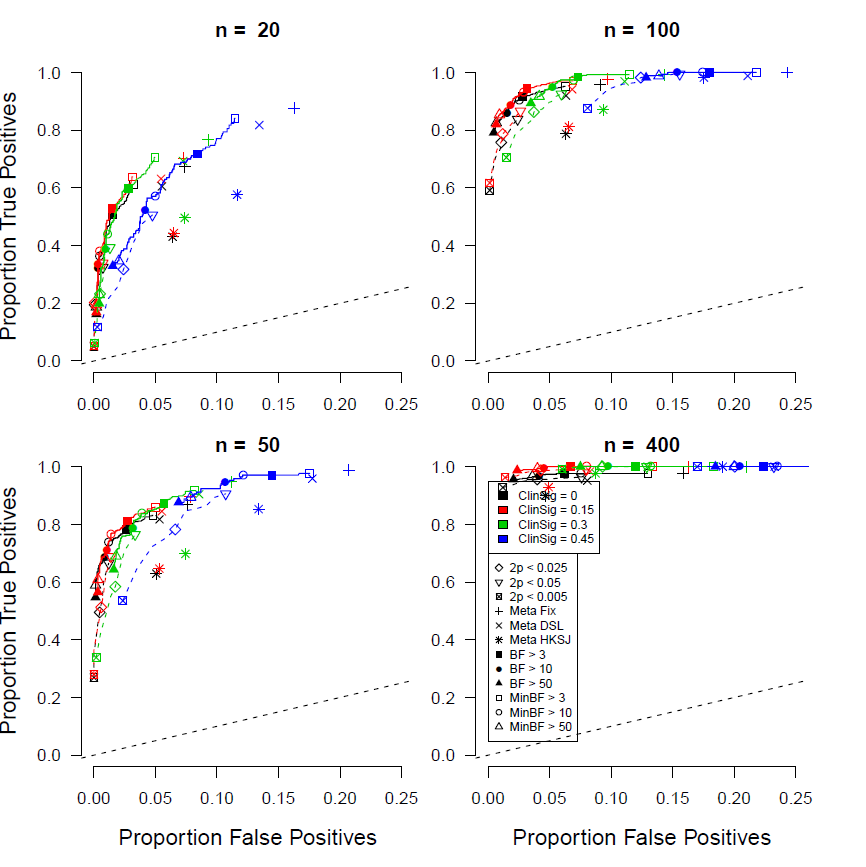
*

*Figure S19*

*
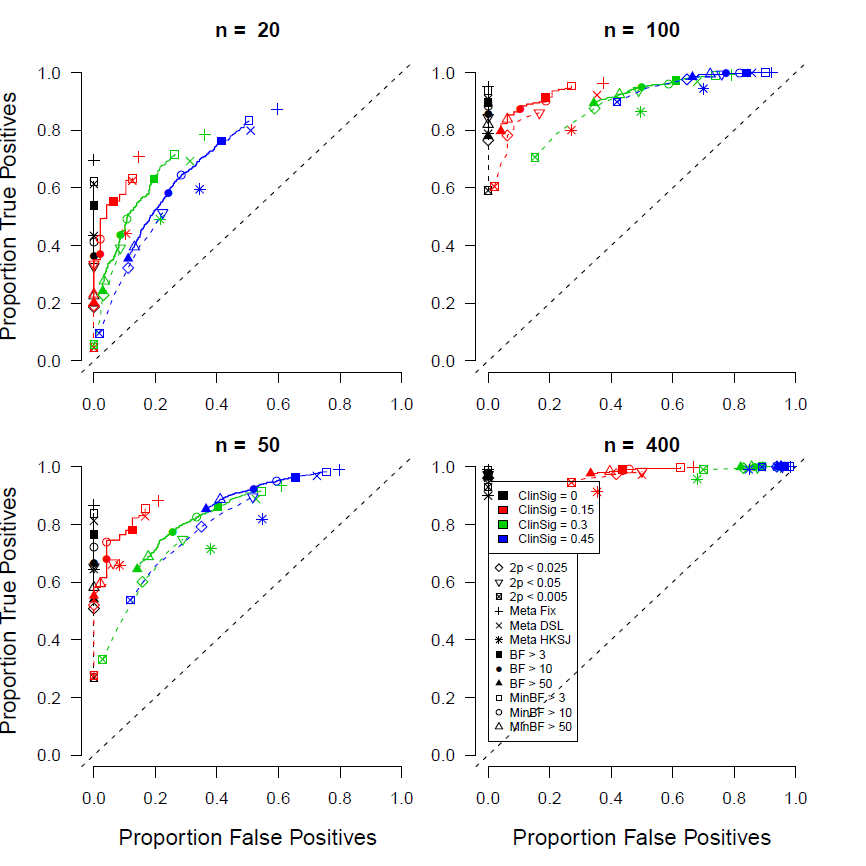
*

*Figure S20*

*
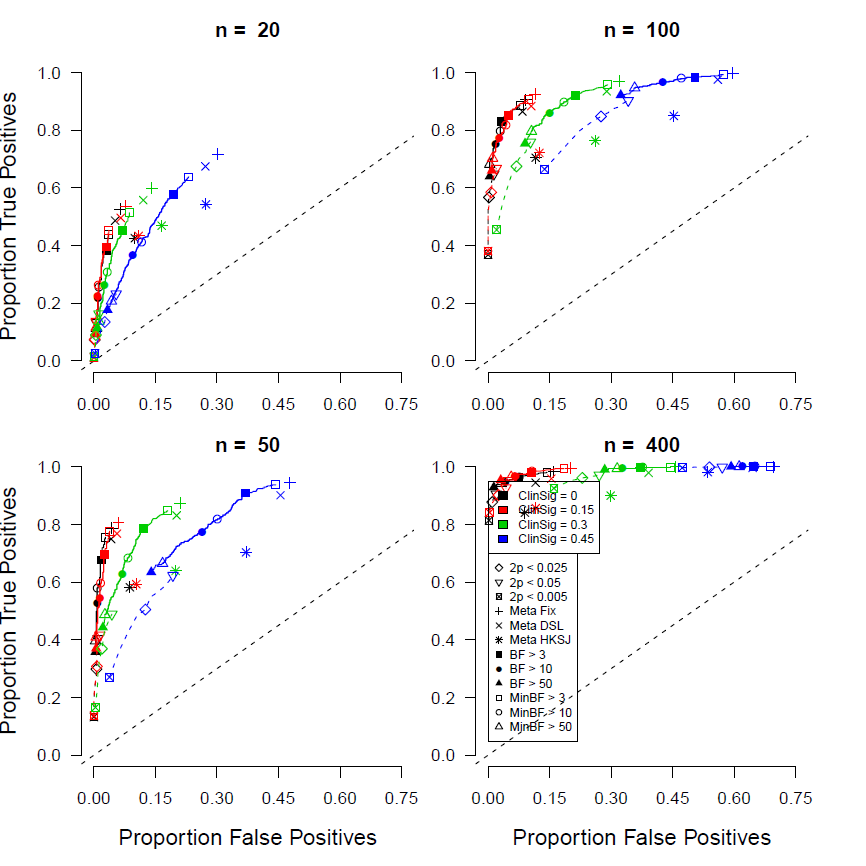
*

*Figure S21*

*
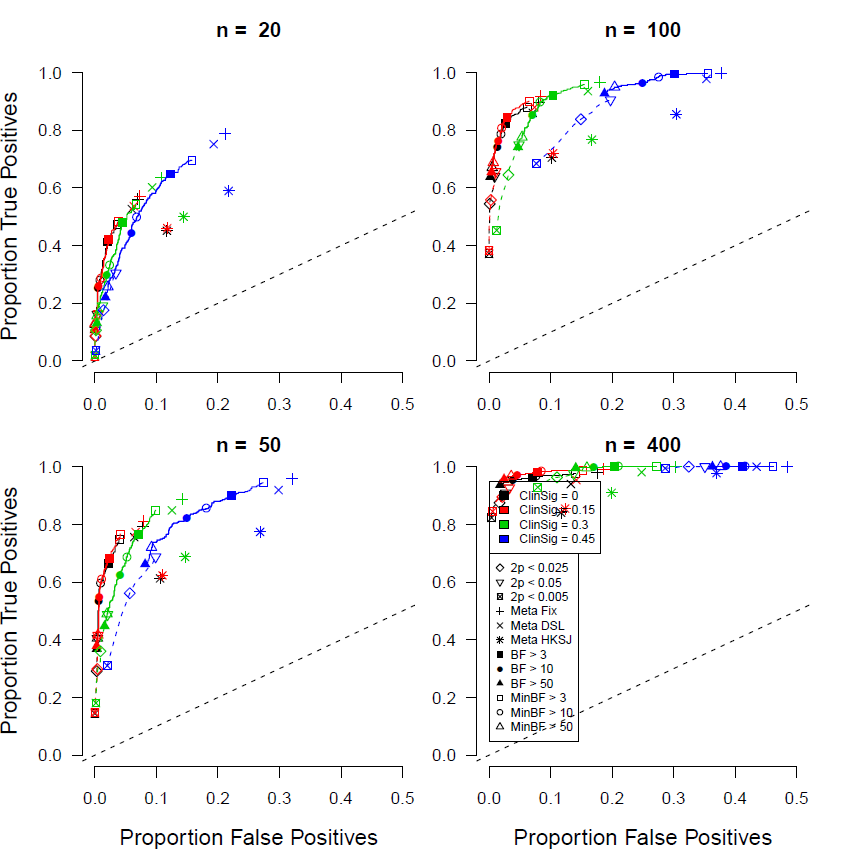
*

*Figure S22*

*
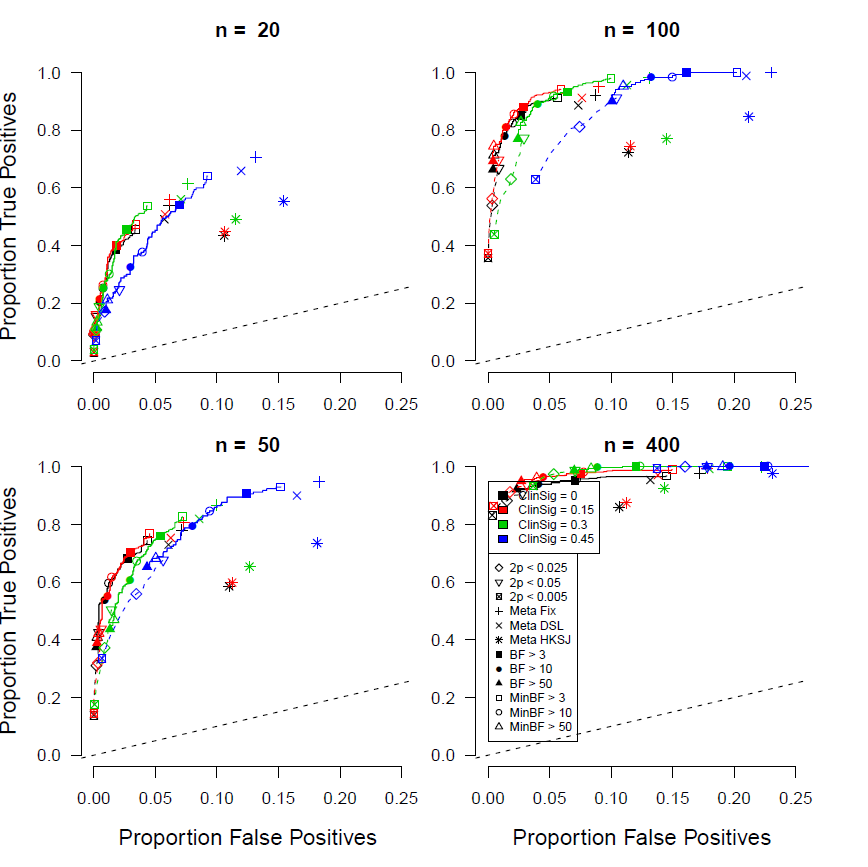
*

*Figure S23*

*
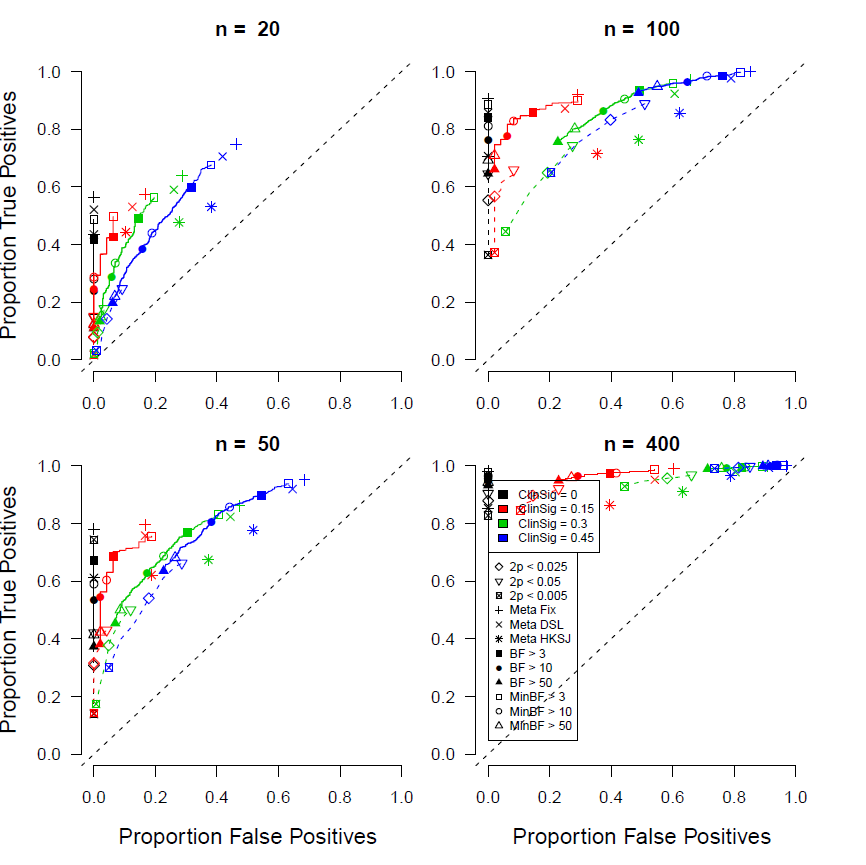
*

*Figure S24*
